# Cod-Liver Oil as a Therapeutic Agent

**Published:** 1876-08

**Authors:** 


Cod-liver Oil as a Therapeutic Agent.—In the *Transactions of the State Medical Society of Arkansas*, Dr. T. W. Hurley writes:

Cod-liver oil, as stated, contains iodine, bromine, etc., but does it contain those substances in sufficient quantity to bring about the happy results claimed by its advocates? Most assuredly not. Then the question naturally arises, What is it in cod-liver oil that renders it a more valuable therapeutic agent than any other oleaginous substance? I answer most emphatically, Nothing; its digestion and assimilation being precisely that of other oleaginous substances.

Several distinguished authors assert that analogy favors the supposition that other animal and vegetable oils, if purified, would be no less eligible, but would possess an obvious advantage in being more abundant and less expensive. What property is common to cod-liver and other oils that produce these analogous effects? The question, it does seem to me, naturally suggests the answer, viz: The property to mystify. Some will, doubtless, say its general constitutents are such that no other oil, or fat, or artificial combination can be had that will accomplish the same results; that to the nutritive properties of the oil are superadded the alterative and stimulating powers of iodine, bromine, etc.; therefore the efficacy of cod-liver oil cannot be doubted. This is simply a begging of the question, and does not even amount to an affirmative. No well-informed man, possessing skill, will wed himself to a remedy that may have acquired notoriety as a specific, while it is endowed with no such quality.

Divest cod-liver oil of its nutrient properties, and it does not possess a single virtue not found in other animal oils not derived from the liver, and some of the vegetable oils also. Neither are the nutrient properties of cod-liver oil superior to those of other oils. How often does the oil prove indigestible, giving rise to nausea and disgusting eructations? The majority of patients have such a repugnance for it that it cannot be overcome by any combination to mask it. It seems quite extraordinary that, notwithstanding the disgusting qualities of the oil, in many instances these are its chief recommendations.

I have no confidence in any specific effect of cod-liver oil in any of the various diseases for which it is recommended.

Experience has already demonstrated to me that any good effect that may, in any given case, result from its use arises from the patient having an excellent duodenal digestion, and hence a proper assimilation as an article of food; but that in a great majority of cases, instead of being digested and properly assimilated, it deranges that catalytic process in which it should legitimately play no part. This oil holds its rank with many physicians who have long been accustomed to prescribe it in many intractible varieties of constitutional affections, but it can truly be said to have wholly lost caste with others. But this fact cannot be attributed alone to its variableness in quality, or adulterations. Observation of facts and a more careful study of its therapeutic agency have wrought the change.—*Half-Yearly Compendium of Medical Science.*

